# Dataset of quantitative structured office measurements of movements in the extremities

**DOI:** 10.1016/j.dib.2020.105876

**Published:** 2020-06-18

**Authors:** Timothy P. Harrigan, Brian J. Hwang, Anil K. Mathur, Kelly A. Mills, Alexander Y. Pantelyat, Jee A. Bang, Alveena B. Syed, Pankhuri Vyas, Samuel D. Martin, Armaan Jamal, Liran Ziegelman, Manuel E. Hernandez, Dean F. Wong, James Robert Brašić

**Affiliations:** a Research and Exploratory Development, Applied Physics Laboratory, The Johns Hopkins University, Laurel, MD, United States; b Section of High Resolution Brain Positron Emission Tomography Imaging, Division of Nuclear Medicine and Molecular Imaging, The Russell H. Morgan Department of Radiology and Radiological Science, The Johns Hopkins University School of Medicine, Baltimore, MD, United States; c Neuromodulation and Advanced Therapies Clinic, Department of Neurology, The Johns Hopkins University School of Medicine, Baltimore, MD, United States; d Atypical Parkinsonism Center, Department of Neurology, The Johns Hopkins University School of Medicine, Baltimore, MD, United States; e Johns Hopkins Huntington Center of Excellence, Department of Neurology, The Johns Hopkins University School of Medicine, Baltimore, MD, United States; f Department of Otolaryngology-Head and Neck Surgery, The Johns Hopkins University School of Medicine, Baltimore, MD, United States; g Krieger School of Arts and Sciences, The Johns Hopkins University, Baltimore, MD, United States; h Neuroscience Program, College of Liberal Arts and Sciences, University of Illinois at Champaign-Urbana, Champaign-Urbana, IL, United States; i Department of Kinesiology and Community Health, College of Applied Health Sciences, University of Illinois at Champaign-Urbana, Champaign-Urbana, IL, United States

**Keywords:** Diagnoses and examinations, Dyskinesia, Fourier transform, Instrumentation, Pronation, Supination, Tremor, Wavelet transform

## Abstract

A low-cost quantitative structured office measurement of movements in the extremities of people with Parkinson's disease [1,2] was performed on people with Parkinson's disease, multiple system atrophy, and age-matched healthy volunteers. Participants underwent twelve videotaped procedures rated by a trained examiner while connected to four accelerometers [1,2] generating a trace of the three location dimensions expressed as spreadsheets [3,4]. The signals of the five repetitive motion items [1,2] underwent processing to fast Fourier [5] and continuous wavelet transforms [6]. The dataset [7] includes the coding form with scores of the live ratings [1,2], the raw files [3], the converted spreadsheets [4], and the fast Fourier [5] and continuous wavelet transforms [6]. All files are unfiltered.

The data also provide findings suitable to compare and contrast with data obtained by investigators applying the same procedure to other populations. Since this is an inexpensive procedure to quantitatively measure motions in Parkinson's disease and other movement disorders, this will be a valuable resource to colleagues, particularly in underdeveloped regions with limited budgets. The dataset will serve as a template for other investigations to develop novel techniques to facilitate the diagnosis, monitoring, and treatment of Parkinson's disease, other movement disorders, and other nervous and mental conditions. The procedure will provide the basis to obtain objective quantitative measurements of participants in clinical trials of new agents.

Specifications Table

**Table:** 

Subject	Neurology
Specific subject area	Accelerometer
	Assessment
	Engineering
	Extrapyramidal disorders
	Instrumentation
	Mathematical modeling
	Motion measurement
	Movement disorders
	Parkinson's disease
Type of data	Table 1
	Supplementary Tables 2
How data were acquired	Hardware:
	Dell Latitude (E6530) premier laptop [8]
	Small, low power, 3-axis ±3 g accelerometer. ADXL335 [9]
	Three-axis accelerometer evaluation board. EVAL-ADXL335Z [10]
	USB/ethernet data logger system [11]
	USBMAX. Six-foot high-speed USB revision 2.0 shielded MSL cable. 28A WG/2C+24AWG/2C (UL) E305668 type CM 75 °C CSA 204790 [12]
	Woods 55213143 16/2 low voltage lighting cable, 100-feet [13]
	Software:
	Excel [4]
	WinDaq [3]
	Continuous wavelet ransforms [6]
	Fast Fourier transform (fft) [5]
Data format	Raw
	Analyzed
Parameters for data collection	Each participant was administered the items of the coding form for a low-cost quantitative measurement of movements in the extremities of people with Parkinson's disease [1,2]. Although the laterality of the more severely affected side of participants with Parkinson's disease and parkinsonism syndromes was not recorded, the clinical ratings provide objective means to identify the more severely affected side. Output of each of the twelve signals for each of the twelve items was recorded as raw files [3] and converted to spreadsheets [4]. The signals of the five repetitive items [1,2] were processed as fast Fourier [5] and continuous wavelet transforms [6]. All files are unfiltered.
Description of data collection	A low-cost quantitative measurement of movements in the extremities of people with Parkinson's disease [1,2] was performed on each participant. Participants sat comfortably in a straight-back chair with arms. An examiner placed sensors on the extremities of participants. The wires of the sensors were positioned to avoid interference with the movements of the participants. An examiner certified in the administration of the Movement Disorder Society-Sponsored Revision of the Unified Parkinson's Disease Rating Scale (MDS-UPDRS) [2] administered the protocol and scored the live rating. A technologist recorded the output of the instrumentation. A videographer filmed the testing. Participants returned after a week or more for repeat testing. All files are unfiltered.
Data source location	City/Town/Region: Baltimore/Maryland
	Country: USA
	Latitude 9–17′49′' N (39.2970515° N)
	Longitude 076–35′30′' W ( −76.591633° W)
Data accessibility	Repository name: Mendeley Data
	Data identification number: 1
	Direct URL to data: https://doi.org/10.17632/xs8nycxg9v.1,
Related research article	Author's name G.N. McKay, T.P. Harrigan, J.R. Brašić
	Title A low-cost quantitative continuous measurement of movements in the extremities of people with Parkinson's disease
	Journal MethodsX
	DOI https://doi.org/10.1016/j.mex.2018.12.017

## Value of the Data

• These data are useful to compare and contrast with data collected on other populations.
• Clinicians, administrators, policy planners, and investigators will benefit from these data.
• These data provide the foundations to quantitatively measure key characteristics of people with Parkinson's disease and other disorders for diagnostic and therapeutic purposes.
• These data are useful to apply machine learning to identify pathognomonic characteristics of Parkinson's disease and other disorders.
• These data provide the basis for telemedicine to develop means to assess people who may have Parkinson's disease and other disorders in remote and underserved locations.
• These data provide the framework to construct training sets for machine learning to predict the prognosis of people who may have Parkinson's disease and other disorders.

## 1 Data

The data include supplementary tables of the clinical scores for each item of the protocol [1,2] for each testing session of each participant (Supplementary Table 1) and the change scores (retest-test) for those participants who completed two test (test and retest) sessions (Supplementary Table 2).

The dataset [7] contains the coding forms [1], the raw files [3], the spreadsheets [4], and the fast Fourier [5] and amor and bump continuous wavelet transform files [6]. Table 1 provides a sample script to generate the fast Fourier [5] and amor and bump continuous wavelet transform files [6] from the spreadsheets [3].

**Table 1:** Custom script used for loading data and carrying out both discrete fast Fourier transforms [5] and amor and bump continuous wavelet transforms [6] for the test data of participant 0003.

% Read in the Excel spreadsheets made from the WinDaQ files.
Acc=importdata('AccData\20161227_PDMotion_0003 combine sheet.xlsx');
% Set up data file labels for output.
Name1='wlet0003′;
Name4='.jpg';
% Set up offsets and lengths for right and left episodes (numbers of
samples, from observed data in Excel)
% PS = Pronation/Supination
% TT = Toe tap
% FT = Finger Tap
% HM = Hand Motion
% LA = Leg Agility
PSroff=1008;
PSrlen=446;
PSloff=2075;
PSllen=441;
TTroff=861;
TTrlen=326;
TTloff=1932;
TTllen=372;
FTroff=882;
FTrlen=364;
FTloff=2472;
FTllen=244;
HMroff=1140;
HMrlen=426;
HMloff=2194;
HMllen=367;
LAroff=727;
LArlen=413;
LAloff=1672;
LAllen=448;
% Record output of accelerometers one at a time.
% Pronation/supination
% the first three columns are right index finger (x, y, z)
% the second three columns are right wrist (x, y, z)
% the third three columns are left index finger (x, y, z)
% the fourth three columns are left wrist (x, y, z)
Name2='_PS_';
% Set up start (offset) and finish (length) for the right six traces.
sampoff=PSroff;
Len=PSrlen;
sampend=sampoff+Len;
% Copy data.
x1=Acc.data.PS(sampoff:sampend,4);
% Perform fast Fourier transform (FFT).
x1fft=fft(x1);
% Obtain magnitude of transform by taking the absolute value of the complex number.
x1ffta=abs(x1fft);
x2=Acc.data.PS(sampoff:sampend,5);
x2fft=fft(x2);
x2ffta=abs(x2fft);
x3=Acc.data.PS(sampoff:sampend,6);
x3fft=fft(x3);
x3ffta=abs(x3fft);
x4=Acc.data.PS(sampoff:sampend,7);
x4fft=fft(x4);
x4ffta=abs(x4fft);
x5=Acc.data.PS(sampoff:sampend,8);
x5fft=fft(x5);
x5ffta=abs(x5fft);
x6=Acc.data.PS(sampoff:sampend,9);
x6fft=fft(x6);
x6ffta=abs(x6fft);
% Perform bump continuous wavelet transforms and write them out using saveas.
% in Name3 there are three letters.
% r=right, l=left
% f=finger, w=wrist, a=ankle, t=toe
% x, y, and z are components of acceleration.
cwt(x1,'bump',80)
Name3='rfx';
saveas(gcf,strcat(Name1,Name2,Name3,Name4));
cwt(x2,'bump',80)
Name3='rfy';
saveas(gcf,strcat(Name1,Name2,Name3,Name4));
cwt(x3,'bump',80)
Name3='rfz';
saveas(gcf,strcat(Name1,Name2,Name3,Name4));
cwt(x4,'bump',80)
Name3='rwx';
saveas(gcf,strcat(Name1,Name2,Name3,Name4));
cwt(x5,'bump',80)
Name3='rwy';
saveas(gcf,strcat(Name1,Name2,Name3,Name4));
cwt(x6,'bump',80)
Name3='rwz';
saveas(gcf,strcat(Name1,Name2,Name3,Name4));
% Perform amor continuous wavelet transforms and write them out using saveas.
%amor wavelet
cwt(x1,'amor',80)
Name3='rfxA';
saveas(gcf,strcat(Name1,Name2,Name3,Name4));
cwt(x2,'amor',80)
Name3='rfyA';
saveas(gcf,strcat(Name1,Name2,Name3,Name4));
cwt(x3,'amor',80)
Name3='rfzA';
saveas(gcf,strcat(Name1,Name2,Name3,Name4));
cwt(x4,'amor',80)
Name3='rwxA';
saveas(gcf,strcat(Name1,Name2,Name3,Name4));
cwt(x5,'amor',80)
Name3='rwyA';
saveas(gcf,strcat(Name1,Name2,Name3,Name4));
cwt(x6,'amor',80)
Name3='rwzA';
saveas(gcf,strcat(Name1,Name2,Name3,Name4));
% FFTs of the left components (Finger and wrist order are the same.)
% left offset
sampoff=PSloff;
Len=PSllen;
sampend=sampoff+Len;
x7=Acc.data.PS(sampoff:sampend,10);
x7fft=fft(x7);
x7ffta=abs(x7fft);
x8=Acc.data.PS(sampoff:sampend,11);
x8fft=fft(x8);
x8ffta=abs(x8fft);
x9=Acc.data.PS(sampoff:sampend,12);
x9fft=fft(x9);
x9ffta=abs(x9fft);
x10=Acc.data.PS(sampoff:sampend,13);
x10fft=fft(x10);
x10ffta=abs(x10fft);
x11=Acc.data.PS(sampoff:sampend,14);
x11fft=fft(x11);
x11ffta=abs(x11fft);
x12=Acc.data.PS(sampoff:sampend,15);
x12fft=fft(x12);
x12ffta=abs(x12fft);
% Perform bump continuous wavelet transforms and write them out using saveas.
cwt(x7,'bump',80)
Name3='lfx';
saveas(gcf,strcat(Name1,Name2,Name3,Name4));
cwt(x8,'bump',80)
Name3='lfy';
saveas(gcf,strcat(Name1,Name2,Name3,Name4));
cwt(x9,'bump',80)
Name3='lfz';
saveas(gcf,strcat(Name1,Name2,Name3,Name4));
cwt(x10,'bump',80)
Name3='lwx';
saveas(gcf,strcat(Name1,Name2,Name3,Name4));
cwt(x11,'bump',80)
Name3='lwy';
saveas(gcf,strcat(Name1,Name2,Name3,Name4));
cwt(x12,'bump',80)
Name3='lwz';
saveas(gcf,strcat(Name1,Name2,Name3,Name4));
%amor wavelet
% % Perform amor continuous wavelet transforms and write them out using saveas.
cwt(x7,'amor',80)
Name3='lfxA';
saveas(gcf,strcat(Name1,Name2,Name3,Name4));
cwt(x8,'amor',80)
Name3='lfyA';
saveas(gcf,strcat(Name1,Name2,Name3,Name4));
cwt(x9,'amor',80)
Name3='lfzA';
saveas(gcf,strcat(Name1,Name2,Name3,Name4));
cwt(x10,'amor',80)
Name3='lwxA';
saveas(gcf,strcat(Name1,Name2,Name3,Name4));
cwt(x11,'amor',80)
Name3='lwyA';
saveas(gcf,strcat(Name1,Name2,Name3,Name4));
cwt(x12,'amor',80)
Name3='lwzA';
saveas(gcf,strcat(Name1,Name2,Name3,Name4));
% Store FFT data for writing to an Excel file.
PSFFT(1:256,1)=x1ffta(1:256);
PSFFT(1:256,2)=x2ffta(1:256);
PSFFT(1:256,3)=x3ffta(1:256);
PSFFT(1:256,4)=x4ffta(1:256);
PSFFT(1:256,5)=x5ffta(1:256);
PSFFT(1:256,6)=x6ffta(1:256);
PSFFT(1:256,7)=x7ffta(1:256);
PSFFT(1:256,8)=x8ffta(1:256);
PSFFT(1:256,9)=x9ffta(1:256);
PSFFT(1:256,10)=x10ffta(1:256);
PSFFT(1:256,11)=x11ffta(1:256);
PSFFT(1:256,12)=x12ffta(1:256);
% Toe tapping data
% the first 3 columns are right ankle (x, y, z)
% the second 3 columns are right big toe (x, y, z)
% the third 3 columns are left ankle (x, y, z)
% the fourth 3 columns are left big toe (x, y, z)
Name2='_TT_';
sampoff=TTroff;
Len=TTrlen;
sampend=sampoff+Len;
x1=Acc.data.TT(sampoff:sampend,4);
x1fft=fft(x1);
x1ffta=abs(x1fft);
x2=Acc.data.TT(sampoff:sampend,5);
x2fft=fft(x2);
x2ffta=abs(x2fft);
x3=Acc.data.TT(sampoff:sampend,6);
x3fft=fft(x3);
x3ffta=abs(x3fft);
x4=Acc.data.TT(sampoff:sampend,7);
x4fft=fft(x4);
x4ffta=abs(x4fft);
x5=Acc.data.TT(sampoff:sampend,8);
x5fft=fft(x5);
x5ffta=abs(x5fft);
x6=Acc.data.TT(sampoff:sampend,9);
x6fft=fft(x6);
x6ffta=abs(x6fft);
% Perform bump continuous wavelet transforms and write them out using saveas.
cwt(x1,'bump',80)
Name3='rax';
saveas(gcf,strcat(Name1,Name2,Name3,Name4));
cwt(x2,'bump',80)
Name3='ray';
saveas(gcf,strcat(Name1,Name2,Name3,Name4));
cwt(x3,'bump',80)
Name3='raz';
saveas(gcf,strcat(Name1,Name2,Name3,Name4));
cwt(x4,'bump',80)
Name3='rtx';
saveas(gcf,strcat(Name1,Name2,Name3,Name4));
cwt(x5,'bump',80)
Name3='rty';
saveas(gcf,strcat(Name1,Name2,Name3,Name4));
cwt(x6,'bump',80)
Name3='rtz';
saveas(gcf,strcat(Name1,Name2,Name3,Name4));
% Perform amor continuous wavelet transforms and write them out using saveas.
cwt(x1,'amor',80)
Name3='raxA';
saveas(gcf,strcat(Name1,Name2,Name3,Name4));
cwt(x2,'amor',80)
Name3='rayA';
saveas(gcf,strcat(Name1,Name2,Name3,Name4));
cwt(x3,'amor',80)
Name3='razA';
saveas(gcf,strcat(Name1,Name2,Name3,Name4));
cwt(x4,'amor',80)
Name3='rtxA';
saveas(gcf,strcat(Name1,Name2,Name3,Name4));
cwt(x5,'amor',80)
Name3='rtyA';
saveas(gcf,strcat(Name1,Name2,Name3,Name4));
cwt(x6,'amor',80)
Name3='rtzA';
saveas(gcf,strcat(Name1,Name2,Name3,Name4));
% left offset
sampoff=TTloff;
Len=TTllen;
sampend=sampoff+Len;
x7=Acc.data.TT(sampoff:sampend,10);
x7fft=fft(x7);
x7ffta=abs(x7fft);
x8=Acc.data.TT(sampoff:sampend,11);
x8fft=fft(x8);
x8ffta=abs(x8fft);
x9=Acc.data.TT(sampoff:sampend,12);
x9fft=fft(x9);
x9ffta=abs(x9fft);
x10=Acc.data.TT(sampoff:sampend,13);
x10fft=fft(x10);
x10ffta=abs(x10fft);
x11=Acc.data.TT(sampoff:sampend,14);
x11fft=fft(x11);
x11ffta=abs(x11fft);
x12=Acc.data.TT(sampoff:sampend,15);
x12fft=fft(x12);
x12ffta=abs(x12fft);
% Perform bump continuous wavelet transforms and write them out using saveas.
cwt(x7,'bump',80)
Name3='lax';
saveas(gcf,strcat(Name1,Name2,Name3,Name4));
cwt(x8,'bump',80)
Name3='lay';
saveas(gcf,strcat(Name1,Name2,Name3,Name4));
cwt(x9,'bump',80)
Name3='laz';
saveas(gcf,strcat(Name1,Name2,Name3,Name4));
cwt(x10,'bump',80)
Name3='ltx';
saveas(gcf,strcat(Name1,Name2,Name3,Name4));
cwt(x11,'bump',80)
Name3='lty';
saveas(gcf,strcat(Name1,Name2,Name3,Name4));
cwt(x12,'bump',80)
Name3='ltz';
saveas(gcf,strcat(Name1,Name2,Name3,Name4));
% Perform amor continuous wavelet transforms and write them out using saveas.
%amor wavelet
cwt(x7,'amor',80)
Name3='laxA';
saveas(gcf,strcat(Name1,Name2,Name3,Name4));
cwt(x8,'amor',80)
Name3='layA';
saveas(gcf,strcat(Name1,Name2,Name3,Name4));
cwt(x9,'amor',80)
Name3='lazA';
saveas(gcf,strcat(Name1,Name2,Name3,Name4));
cwt(x10,'amor',80)
Name3='ltxA';
saveas(gcf,strcat(Name1,Name2,Name3,Name4));
cwt(x11,'amor',80)
Name3='ltyA';
saveas(gcf,strcat(Name1,Name2,Name3,Name4));
cwt(x12,'amor',80)
Name3='ltzA';
saveas(gcf,strcat(Name1,Name2,Name3,Name4));
%save FFTs in an array for writing
TTFFT(1:256,1)=x1ffta(1:256);
TTFFT(1:256,2)=x2ffta(1:256);
TTFFT(1:256,3)=x3ffta(1:256);
TTFFT(1:256,4)=x4ffta(1:256);
TTFFT(1:256,5)=x5ffta(1:256);
TTFFT(1:256,6)=x6ffta(1:256);
TTFFT(1:256,7)=x7ffta(1:256);
TTFFT(1:256,8)=x8ffta(1:256);
TTFFT(1:256,9)=x9ffta(1:256);
TTFFT(1:256,10)=x10ffta(1:256);
TTFFT(1:256,11)=x11ffta(1:256);
TTFFT(1:256,12)=x12ffta(1:256);
% the rest of the script has the same structure
% finger tap
Name2='_FT_';
sampoff=FTroff;
Len=FTrlen;
sampend=sampoff+Len;
x1=Acc.data.FT(sampoff:sampend,4);
x1fft=fft(x1);
x1ffta=abs(x1fft);
x2=Acc.data.FT(sampoff:sampend,5);
x2fft=fft(x2);
x2ffta=abs(x2fft);
x3=Acc.data.FT(sampoff:sampend,6);
x3fft=fft(x3);
x3ffta=abs(x3fft);
x4=Acc.data.FT(sampoff:sampend,7);
x4fft=fft(x4);
x4ffta=abs(x4fft);
x5=Acc.data.FT(sampoff:sampend,8);
x5fft=fft(x5);
x5ffta=abs(x5fft);
x6=Acc.data.FT(sampoff:sampend,9);
x6fft=fft(x6);
x6ffta=abs(x6fft);
% Perform bump continuous wavelet transforms and write them out using saveas.
cwt(x1,'bump',80)
Name3='rfx';
saveas(gcf,strcat(Name1,Name2,Name3,Name4));
cwt(x2,'bump',80)
Name3='rfy';
saveas(gcf,strcat(Name1,Name2,Name3,Name4));
cwt(x3,'bump',80)
Name3='rfz';
saveas(gcf,strcat(Name1,Name2,Name3,Name4));
cwt(x4,'bump',80)
Name3='rwx';
saveas(gcf,strcat(Name1,Name2,Name3,Name4));
cwt(x5,'bump',80)
Name3='rwy';
saveas(gcf,strcat(Name1,Name2,Name3,Name4));
cwt(x6,'bump',80)
Name3='rwz';
saveas(gcf,strcat(Name1,Name2,Name3,Name4));
% Perform amor continuous wavelet transforms and write them out using saveas.
cwt(x1,'amor',80)
Name3='rfxA';
saveas(gcf,strcat(Name1,Name2,Name3,Name4));
cwt(x2,'amor',80)
Name3='rfyA';
saveas(gcf,strcat(Name1,Name2,Name3,Name4));
cwt(x3,'amor',80)
Name3='rfzA';
saveas(gcf,strcat(Name1,Name2,Name3,Name4));
cwt(x4,'amor',80)
Name3='rwxA';
saveas(gcf,strcat(Name1,Name2,Name3,Name4));
cwt(x5,'amor',80)
Name3='rwyA';
saveas(gcf,strcat(Name1,Name2,Name3,Name4));
cwt(x6,'amor',80)
Name3='rwzA';
saveas(gcf,strcat(Name1,Name2,Name3,Name4));
sampoff=FTloff;
Len=FTllen;
sampend=sampoff+Len;
x7=Acc.data.FT(sampoff:sampend,10);
x7fft=fft(x7);
x7ffta=abs(x7fft);
x8=Acc.data.FT(sampoff:sampend,11);
x8fft=fft(x8);
x8ffta=abs(x8fft);
x9=Acc.data.FT(sampoff:sampend,12);
x9fft=fft(x9);
x9ffta=abs(x9fft);
x10=Acc.data.FT(sampoff:sampend,13);
x10fft=fft(x10);
x10ffta=abs(x10fft);
x11=Acc.data.FT(sampoff:sampend,14);
x11fft=fft(x11);
x11ffta=abs(x11fft);
x12=Acc.data.FT(sampoff:sampend,15);
x12fft=fft(x12);
x12ffta=abs(x12fft);
% Perform bump continuous wavelet transforms and write them out using saveas.
cwt(x7,'bump',80)
Name3='lfx';
saveas(gcf,strcat(Name1,Name2,Name3,Name4));
cwt(x8,'bump',80)
Name3='lfy';
saveas(gcf,strcat(Name1,Name2,Name3,Name4));
cwt(x9,'bump',80)
Name3='lfz';
saveas(gcf,strcat(Name1,Name2,Name3,Name4));
cwt(x10,'bump',80)
Name3='lwx';
saveas(gcf,strcat(Name1,Name2,Name3,Name4));
cwt(x11,'bump',80)
Name3='lwy';
saveas(gcf,strcat(Name1,Name2,Name3,Name4));
cwt(x12,'bump',80)
Name3='lwz';
saveas(gcf,strcat(Name1,Name2,Name3,Name4));
% Perform amor continuous wavelet transforms and write them out using saveas.
cwt(x7,'amor',80)
Name3='lfxA';
saveas(gcf,strcat(Name1,Name2,Name3,Name4));
cwt(x8,'amor',80)
Name3='lfyA';
saveas(gcf,strcat(Name1,Name2,Name3,Name4));
cwt(x9,'amor',80)
Name3='lfzA';
saveas(gcf,strcat(Name1,Name2,Name3,Name4));
cwt(x10,'amor',80)
Name3='lwxA';
saveas(gcf,strcat(Name1,Name2,Name3,Name4));
cwt(x11,'amor',80)
Name3='lwyA';
saveas(gcf,strcat(Name1,Name2,Name3,Name4));
cwt(x12,'amor',80)
Name3='lwzA';
saveas(gcf,strcat(Name1,Name2,Name3,Name4));
FTFFT(1:256,1)=x1ffta(1:256);
FTFFT(1:256,2)=x2ffta(1:256);
FTFFT(1:256,3)=x3ffta(1:256);
FTFFT(1:256,4)=x4ffta(1:256);
FTFFT(1:256,5)=x5ffta(1:256);
FTFFT(1:256,6)=x6ffta(1:256);
FTFFT(1:256,7)=x7ffta(1:256);
FTFFT(1:256,8)=x8ffta(1:256);
FTFFT(1:256,9)=x9ffta(1:256);
FTFFT(1:256,10)=x10ffta(1:256);
FTFFT(1:256,11)=x11ffta(1:256);
FTFFT(1:256,12)=x12ffta(1:256);
%Hand motion
Name2='_HM_';
sampoff=HMroff;
Len=HMrlen;
sampend=Len+sampoff;
x1=Acc.data.HM(sampoff:sampend,4);
x1fft=fft(x1);
x1ffta=abs(x1fft);
x2=Acc.data.HM(sampoff:sampend,5);
x2fft=fft(x2);
x2ffta=abs(x2fft);
x3=Acc.data.HM(sampoff:sampend,6);
x3fft=fft(x3);
x3ffta=abs(x3fft);
x4=Acc.data.HM(sampoff:sampend,7);
x4fft=fft(x4);
x4ffta=abs(x4fft);
x5=Acc.data.HM(sampoff:sampend,8);
x5fft=fft(x5);
x5ffta=abs(x5fft);
x6=Acc.data.HM(sampoff:sampend,9);
x6fft=fft(x6);
x6ffta=abs(x6fft);
% Perform amor continuous wavelet transforms and write them out using saveas.
cwt(x1,'amor',80)
Name3='rfxA';
saveas(gcf,strcat(Name1,Name2,Name3,Name4));
cwt(x2,'amor',80)
Name3='rfyA';
saveas(gcf,strcat(Name1,Name2,Name3,Name4));
cwt(x3,'amor',80)
Name3='rfzA';
saveas(gcf,strcat(Name1,Name2,Name3,Name4));
cwt(x4,'amor',80)
Name3='rwxA';
saveas(gcf,strcat(Name1,Name2,Name3,Name4));
cwt(x5,'amor',80)
Name3='rwyA';
saveas(gcf,strcat(Name1,Name2,Name3,Name4));
cwt(x6,'amor',80)
Name3='rwzA';
saveas(gcf,strcat(Name1,Name2,Name3,Name4));
sampoff=HMloff;
len=HMllen;
sampend=len+sampoff;
x7=Acc.data.HM(sampoff:sampend,10);
x7fft=fft(x7);
x7ffta=abs(x7fft);
x8=Acc.data.HM(sampoff:sampend,11);
x8fft=fft(x8);
x8ffta=abs(x8fft);
x9=Acc.data.HM(sampoff:sampend,12);
x9fft=fft(x9);
x9ffta=abs(x9fft);
x10=Acc.data.HM(sampoff:sampend,13);
x10fft=fft(x10);
x10ffta=abs(x10fft);
x11=Acc.data.HM(sampoff:sampend,14);
x11fft=fft(x11);
x11ffta=abs(x11fft);
x12=Acc.data.HM(sampoff:sampend,15);
x12fft=fft(x12);
x12ffta=abs(x12fft);
% Perform bump continuous wavelet transforms and write them out using saveas.
cwt(x7,'bump',80)
Name3='lfx';
saveas(gcf,strcat(Name1,Name2,Name3,Name4));
cwt(x8,'bump',80)
Name3='lfy';
saveas(gcf,strcat(Name1,Name2,Name3,Name4));
cwt(x9,'bump',80)
Name3='lfz';
saveas(gcf,strcat(Name1,Name2,Name3,Name4));
cwt(x10,'bump',80)
Name3='lwx';
saveas(gcf,strcat(Name1,Name2,Name3,Name4));
cwt(x11,'bump',80)
Name3='lwy';
saveas(gcf,strcat(Name1,Name2,Name3,Name4));
cwt(x12,'bump',80)
Name3='lwz';
saveas(gcf,strcat(Name1,Name2,Name3,Name4));
% Perform amor continuous wavelet transforms and write them out using saveas.
cwt(x7,'amor',80)
Name3='lfxA';
saveas(gcf,strcat(Name1,Name2,Name3,Name4));
cwt(x8,'amor',80)
Name3='lfyA';
saveas(gcf,strcat(Name1,Name2,Name3,Name4));
cwt(x9,'amor',80)
Name3='lfzA';
saveas(gcf,strcat(Name1,Name2,Name3,Name4));
cwt(x10,'amor',80)
Name3='lwxA';
saveas(gcf,strcat(Name1,Name2,Name3,Name4));
cwt(x11,'amor',80)
Name3='lwyA';
saveas(gcf,strcat(Name1,Name2,Name3,Name4));
cwt(x12,'amor',80)
Name3='lwzA';
saveas(gcf,strcat(Name1,Name2,Name3,Name4));
HMFFT(1:256,1)=x1ffta(1:256);
HMFFT(1:256,2)=x2ffta(1:256);
HMFFT(1:256,3)=x3ffta(1:256);
HMFFT(1:256,4)=x4ffta(1:256);
HMFFT(1:256,5)=x5ffta(1:256);
HMFFT(1:256,6)=x6ffta(1:256);
HMFFT(1:256,7)=x7ffta(1:256);
HMFFT(1:256,8)=x8ffta(1:256);
HMFFT(1:256,9)=x9ffta(1:256);
HMFFT(1:256,10)=x10ffta(1:256);
HMFFT(1:256,11)=x11ffta(1:256);
HMFFT(1:256,12)=x12ffta(1:256);
%Leg Agility
Name2='_LA_';
sampoff=LAroff;
len=LArlen;
sampend=len+sampoff;
x1=Acc.data.LA(sampoff:sampend,4);
x1fft=fft(x1);
x1ffta=abs(x1fft);
x2=Acc.data.LA(sampoff:sampend,5);
x2fft=fft(x2);
x2ffta=abs(x2fft);
x3=Acc.data.LA(sampoff:sampend,6);
x3fft=fft(x3);
x3ffta=abs(x3fft);
x4=Acc.data.LA(sampoff:sampend,7);
x4fft=fft(x4);
x4ffta=abs(x4fft);
x5=Acc.data.LA(sampoff:sampend,8);
x5fft=fft(x5);
x5ffta=abs(x5fft);
x6=Acc.data.LA(sampoff:sampend,9);
x6fft=fft(x6);
x6ffta=abs(x6fft);
% Perform bump continuous wavelet transforms and write them out using saveas.
cwt(x1,'bump',80)
Name3='rax';
saveas(gcf,strcat(Name1,Name2,Name3,Name4));
cwt(x2,'bump',80)
Name3='ray';
saveas(gcf,strcat(Name1,Name2,Name3,Name4));
cwt(x3,'bump',80)
Name3='raz';
saveas(gcf,strcat(Name1,Name2,Name3,Name4));
cwt(x4,'bump',80)
Name3='rtx';
saveas(gcf,strcat(Name1,Name2,Name3,Name4));
cwt(x5,'bump',80)
Name3='rty';
saveas(gcf,strcat(Name1,Name2,Name3,Name4));
cwt(x6,'bump',80)
Name3='rtz';
saveas(gcf,strcat(Name1,Name2,Name3,Name4));
Perform amor continuous wavelet transforms and write them out using saveas.
cwt(x1,'amor',80)
Name3='raxA';
saveas(gcf,strcat(Name1,Name2,Name3,Name4));
cwt(x2,'amor',80)
Name3='rayA';
saveas(gcf,strcat(Name1,Name2,Name3,Name4));
cwt(x3,'amor',80)
Name3='razA';
saveas(gcf,strcat(Name1,Name2,Name3,Name4));
cwt(x4,'amor',80)
Name3='rtxA';
saveas(gcf,strcat(Name1,Name2,Name3,Name4));
cwt(x5,'amor',80)
Name3='rtyA';
saveas(gcf,strcat(Name1,Name2,Name3,Name4));
cwt(x6,'amor',80)
Name3='rtzA';
saveas(gcf,strcat(Name1,Name2,Name3,Name4));
sampoff=LAloff;
len=LAllen;
sampend=len+sampoff;
x7=Acc.data.LA(sampoff:sampend,10);
x7fft=fft(x7);
x7ffta=abs(x7fft);
x8=Acc.data.LA(sampoff:sampend,11);
x8fft=fft(x8);
x8ffta=abs(x8fft);
x9=Acc.data.LA(sampoff:sampend,12);
x9fft=fft(x9);
x9ffta=abs(x9fft);
x10=Acc.data.LA(sampoff:sampend,13);
x10fft=fft(x10);
x10ffta=abs(x10fft);
x11=Acc.data.LA(sampoff:sampend,14);
x11fft=fft(x11);
x11ffta=abs(x11fft);
x12=Acc.data.LA(sampoff:sampend,15);
x12fft=fft(x12);
x12ffta=abs(x12fft);
% Perform bump continuous wavelet transforms and write them out using saveas.
cwt(x7,'bump',80)
Name3='lax';
saveas(gcf,strcat(Name1,Name2,Name3,Name4));
cwt(x8,'bump',80)
Name3='lay';
saveas(gcf,strcat(Name1,Name2,Name3,Name4));
cwt(x9,'bump',80)
Name3='laz';
saveas(gcf,strcat(Name1,Name2,Name3,Name4));
cwt(x10,'bump',80)
Name3='ltx';
saveas(gcf,strcat(Name1,Name2,Name3,Name4));
cwt(x11,'bump',80)
Name3='lty';
saveas(gcf,strcat(Name1,Name2,Name3,Name4));
cwt(x12,'bump',80)
Name3='ltz';
saveas(gcf,strcat(Name1,Name2,Name3,Name4));
% Perform amor continuous wavelet transforms and write them out using saveas.
cwt(x7,'amor',80)
Name3='laxA';
saveas(gcf,strcat(Name1,Name2,Name3,Name4));
cwt(x8,'amor',80)
Name3='layA';
saveas(gcf,strcat(Name1,Name2,Name3,Name4));
cwt(x9,'amor',80)
Name3='lazA';
saveas(gcf,strcat(Name1,Name2,Name3,Name4));
cwt(x10,'amor',80)
Name3='ltxA';
saveas(gcf,strcat(Name1,Name2,Name3,Name4));
cwt(x11,'amor',80)
Name3='ltyA';
saveas(gcf,strcat(Name1,Name2,Name3,Name4));
cwt(x12,'amor',80)
Name3='ltzA';
saveas(gcf,strcat(Name1,Name2,Name3,Name4));
LAFFT(1:256,1)=x1ffta(1:256);
LAFFT(1:256,2)=x2ffta(1:256);
LAFFT(1:256,3)=x3ffta(1:256);
LAFFT(1:256,4)=x4ffta(1:256);
LAFFT(1:256,5)=x5ffta(1:256);
LAFFT(1:256,6)=x6ffta(1:256);
LAFFT(1:256,7)=x7ffta(1:256);
LAFFT(1:256,8)=x8ffta(1:256);
LAFFT(1:256,9)=x9ffta(1:256);
LAFFT(1:256,10)=x10ffta(1:256);
LAFFT(1:256,11)=x11ffta(1:256);
LAFFT(1:256,12)=x12ffta(1:256);
% Write out the FFT data as a single Excel file with multiple pages.
xlswrite('PDFFT_0003.xlsx',PSFFT,'Pro-Sup');
xlswrite('PDFFT_0003.xlsx',HMFFT,'Hand Motion');
xlswrite('PDFFT_0003.xlsx',FTFFT,'Finger Tap');
xlswrite('PDFFT_0003.xlsx',TTFFT,'Toe Tap');
xlswrite('PDFFT_0003.xlsx',LAFFT,'Leg Agility');

## 2 Experimental design, materials, and methods

The work described has been carried out in accordance with the Code of Ethics of the World Medical Association (Declaration of Helsinki) for experiments involving humans [14]. The manuscript is in line with the Recommendations for the Conduct, Reporting, Editing and Publication of Scholarly Work in Medical Journals of the International Committee of Medical Journal Editors [15]. The equipment was approved for human experimentation by Clinical Engineering at Johns Hopkins Hospital in Baltimore, Maryland. The protocol was approved by the Johns Hopkins Institutional Review Boards, Baltimore, Maryland. Although the specific instrumentation for the study was approved for human investigation by the institution, the authors declare that the use of the instrumentation decribed in this article for humans is unlabeled and unapproved. Investigators must verify that equipment comparable to the current study can be safely used on humans. Written informed consent was obtained from each participant.

*Construction of instrumentation.* Accelerometers [9] attached to evaluation boards [10] connected to shielded cables [13] were taped with paper tape to the extremities of participants. The shielded cables [13] were connected to a data logger [11] within a metal box. The data logger was connected with a shielded cable [12] to a laptop computer [8].

Participants with Parkinson's disease and atypical parkinsonism were recruited from the clinical practices of co-authors who are faculty neurologists specializing in movement disorders at the Johns Hopkins University School of Medicine in Baltimore, Maryland (AYP, JAB, and KAM). Healthy adults from the community were selected to match the sex and age of participants with Parkinson's disease.

Participants underwent the protocol [1,2] with responses scored by trained examiners certified in the administration of the Movement Disorder Society-Sponsored Revision of the Unified Parkinson's Disease Rating Scale (MDS‐UPDRS) [2] while instrumentation recorded the output of the accelerometers [9] attached to the participants. Ten participants with Parkinson's disease and one participant with multiple system atrophy underwent the test procedure once. Ten participants with Parkinson's disease and eight healthy controls underwent a retest of the protocol [1,2] a week or more after the initial testing. Each testing includes twelve procedures [1,2]. Since the initial test was incomplete for one participant with Parkinson's disease, he completed two re-tests. The first retest served as the test session; the second retest served as the retest.

Each procedure was scored on the coding form for a low-cost quantitative measurement of movements in the extremities of people with Parkinson's disease [1,2] in real time by a researcher who was certified in the administration of the Movement Disorder Society‐sponsored revision of the Unified Parkinson's Disease Rating Scale (MDS‐UPDRS) [2]. Participants were seated in a chair with arms and a straight back without wheels. The chair was placed approximately six inches from a wall so that the participant's head would not hit the wall during any of the procedures. Colleagues were placed on either side of participants to catch them in case of falling if necessary due to the impairments of the participants. The examiner was seated in a similar chair facing the participant approximately three feet from the participant. The protocol was usually performed before or after the clinical appointment with the treating neurologist. Therefore, the procedures were performed in multiple locations with family members and the members of the research team in attendance. The examiner circled the specific abnormality in the performance of each task and indicated which side was affected on the coding forms. The completed coding forms are included in the data files for each participant [7].

The instrumentation generated twelve files for the output of each procedure corresponding to the three dimensions of the four accelerometers [9] positioned on the participants for each procedure [1.2]. The raw output from the instrumentation [3] was included in the dataset [7] for each participant. Each raw output file [3] was then converted into spreadsheets [4] included in the dataset [7]. Coding forms [1,2] for the sessions are included in the dataset [7]. The data from the spreadsheets [4] was utilized to obtain fast Fourier [5,^7^,[16], [17], [18], [19], [20], [21], [22] and continuous wavelet transforms [6,^7^,[16], [17], [18], [19], [20], [21], [22].

The scores on the coding forms are tabulated in Supplementary Table 1. The shortest duration between test and retest was a week. If a retest took place less than a month after a test, then the interval between test and retest was recorded as one month. The data in Supplementary Table 1 represent the most accurate representations of the available data. In particular, participant 0001 was erroneously reported to have had deep brain stimulation implanted between test and retest [16]. Careful review of the clinical record reveale that he actually had deep brain stimulation implanted before the test session.

*Measurements of height and weight.* Future analyses will include estimates of the lever arm for the upper and lower extremities. While the length of the forearm and the leg were not measured, the height and the weight of each participant provide foundations to estimate these measurements. With the estimates of the length of the forearm and leg, robotic simulations may be accomplished.

The data of the height and the weight of participants were obtained from the clinical record. Since measurements of height and weight were often not made on the days of the testing sessions, the measurements of height and weight from the date closest to the testing session date were recorded. Sometimes the measurements of height and weight were made years before or after the date of the testing session. When no measurements of height and weight were available, a period was placed the box in Supplementary Table 1 to indicate missing data.

*Measurements of the Movement Disorder Society‐Sponsored Revision of the Unified Parkinson's Disease Rating Scale (MDS‐UPDRS) [**2**].* The examiner performed only the twelve items of the coding form, modified from the MDS-UPDRS [1,2], not the full MDS-UPDRS [2] during each testing session. The full MDS-UPDRS [2] was often performed by the attending neurologist during the clinical visit that often preceded or followed the testing session. The scores of the rating by the clinical neurologist from the clinical record on the date of the testing session were recorded in Supplementary Table 1.

*Measurements of the use and effect of levodopa and deep brain stimulation.* Midway during the project examiners began recording on the coding forms [1,2] (A) if the participant currently used levodopa and if the effect was on and (B) if the participant had the implanted device for deep brain stimulation (DBS) and if the effect was on. The clinical record was used to determine the use and effect of levodopa and DBS for most of the participants (Supplementary Table 1). If levodopa and DBS were used at the time of the testing session, the effect was assumed to be on. Participant 0001 was erroneously reported to have no DBS during the test session [7,16]. Review of the clinical record confirmed that participant 0001 had DBS during both test and retest sessions (Supplementary Table 1). The DBS for participant 0001 was being adjusted on the day of the test session. Optimal DBS adjustment was attained at the time of the retest for participant 0001.

Although participant 16 provided consent for the project, no tests were performed so there is no data. The records of the test sessions of participants 5 and 14 included only items for the upper extremity (3.17 Rest tremor amplitude upper limbs right and left, 3.17 Rest tremor amplitude upper limbs counting right and left, 3.15 Postural tremor of the hands right and left, 3.4 Finger tapping right and left, 3.5 Hand movements right and left, 3.6 Pronation-supination movements of the hands right and left, 3.9  Arising from chair upper limbs) [1,2]. Since the initial test was incomplete, a second retest was performed on participant 14. For participant 14 the first retest may be used as the test and the second retest as the retest. For participant 15 only some items for the upper extremity (3.17 Rest tremor amplitude upper limbs right and left, 3.17 Rest tremor amplitude upper limbs counting right and left, 3.15 Postural tremor of the hands right and left, 3.4 Finger tapping right and left, 3.5 Hand movements right and left) [1,2] were obtained.

For participants who completed full tests and retests change scores (retest – test) were calculated (Supplementary Table 2). Since the initial test for participant 14 contained only the analysis of the upper extremity, it was incomplete. Therefore, the first retest was used as the test and the second retest as the retest for participant 14.

*Analysis of data files.*

*Repetitive movement procedures.*

3.4 Finger tapping [1,2]
3.5 Hand movements [1,2]
3.6 Pronation-supination movements [1,2]
3.7 Toe tapping [1,2]
3.8 Leg agility [1,2]

The time frame of the spreadsheets [4] was reduced to capture only the segments corresponding to the repetitive movements. The segments corresponding to the movements of the right and left extremities were extracted as signal files for analysis for fast Fourier [5] and amor and bump continuous wavelet transforms [6] with scripts (Table 1). Since this is a pilot protocol, amor and bump continuous wavelet transforms [6] were both analyzed to identify unique attributes [7].

Files are identified with T for test, R for retest, R1 for retest 1, R2 for retest 2, T for toe, A for ankle, F for finger, W for wrist, A for amor, and B for bump [7].

All files are unfiltered.

Videotapes of test and retest sessions were obtained for participants 17, 20, 23, 24, and 25.

## 3 Limitations

*Placement of sensors by visual observation, not measurement with a tape measure.* Sensors were placed in the indicated locations by visual estimation without precise measurement of distances on the protocol [1,2].

Future investigations may benefit from precise measurements of the placements of the sensors relative to anatomical landmarks or measurements of segment lengths in order to provide estimates of relevant lever arms. Alternatively, the placement of sensors may be altered for each participant to a particular ratio, say 1:10, from the wrist to the elbow and from the ankle to the knee in order to generate a uniform estimate of the lever arm. Future investigations may benefit from measuring the length of the forearm and the leg on each side of each participant in order to estimate the lever arms.

The measurements of the height and the weight of the individuals obtained (Supplementary Table 1) may provide the bases to estimate the lengths of the forearm and the leg to estimate the lever arms of movements for robotic simulations.

*Ratings of visual observation and signal processing of instrumentation on more than the indicated ten repetitions for repetitive items.* Items requiring ten repetitions (3.4 Finger tapping, 3.5 Hand movements, 3.6 Pronation-supination movements of the hands, 3.7 Toe tapping, 3.8 Leg agility [1,2]) were performed for far more than ten repetitions. Participants were routinely asked to perform the movements as fast and fully as possible after a few repetitions. Then they were recorded for around 60 more repetitions in order to provide adequate data for signal processing of the output of the instrumentation. They were scored on the coding form for the duration of testing, not merely a set of ten repetitions. In other words, although the instructions indicated only ten repetitions for items requiring ten repetitions (3.4 Finger tapping, 3.5 Hand movements, 3.6 Pronation-supination movements of the hands, 3.7 Toe tapping, 3.8 Leg agility [1,2]), up to around 60 repetitions were routinely recorded and scored. None of the sessions were limited to only ten repetitions. Correlation of videotapes with output of instrumentation will provide the bases to accurately limit the assessments to ten repetitions. Examiners may benefit from identifying an optimal sequence of ten repetitions. Examiners may then limit the scoring on the coding form of the videotape and the signal analysis of instrumentation output to the specific optimal sequence of ten repetitions.

*Need for reliability of assessments.* The ratings were performed by raters who were certified in the administration of the MDS-UDPRS [2]. Ratings were performed live in the original testing session by a single rater. Although test and retest were routinely performed by the same rater for individual participants, occasionally the raters were different for the test and retest sessions. Testing sessions were performed at multiple locations typically near the clinical visits of the participants. Further investigations may benefit from conducting ratings independently by multiple raters in the live session and in videotape analysis to assess the interrater reliability of the raters. The uniformity of future studies may be enhanced by the performance of all ratings by the same individual or team of raters. Test-retest reliability of ratings may be strengthened by conducting all tests and retests by a single examiner in a single specified location with the same temperature-controlled environment at the same time of day during a specific time period, say a week or a month, after the test. Reliability may also be enhanced by rating videos of the sessions. Additionally, performance by the same examiner of the full MDS-UDPRS [2] consistently either before or after the testing sessions will enhance the validity of those measurements. In other words regular peer review can enhance and sustain the interrater reliability of individual raters. A video may be played frame by frame with a toggle switch to view each movement. Also, video segments may be replayed to confirm initial impressions.

Measuring the height, the weight, and the forearm lengths of each participant consistently either before or after each testing session will ensure the accuracy of those measurements. The coding form for a low-cost quantitative measurement of movements in the extremities of people with Parkinson's disease [1] can be improved to contain measurements of the height, the weight, and the forearm length of participants for the estimation of biomechanical parameters.

*Need to record treatment status of participants.* Midway during the conduct of the experiment, the examiner started to record if participants (A) took levodopa and if the levodopa effect was on or off and if participants had deep brain stimulation (DBS) and if the DBS effect was on or off. This data will be recorded with every administration of the coding form [1,2]. Therefore, the levodopa and DBS status of each participant for each testing will be recorded in the future.

*Need to record specific reasons for scores of items of participants.* Midway during the conduct of the experiment, the examiner started to record the specific reasons for scores including laterality for each live rating. Now this data is recorded with every administration of the coding form [1,2]. By means of the handwritten notes on the coding forms, the examiner records if the score is due to the occurrences of (a) interruptions, halts, or freezes, (b) slowing, and (c) decrements in amplitude [1], [2]. Including this information in the summary table (Supplementary Table 1) will enhance the ability of scores to identify the precise abnormalities observed by the examiner during the rating session. Due to the absence of notations on forms early in the study and to the uncertainties of interpreting the notes of the raters [7], the current scores are provided without the specifications of a, b, or c to indicate the type of abnormality. However, future analyses of the dataset [7] may be enhanced by providing best estimates of the meaning of the notes on the coding forms [7]. Therefore, the specific reasons for scores of each participant for each testing will be recorded in the future.

*Need to record the laterality of the more affected extremity of participants with Parkinson's disease.* Since the symptoms and signs of Parkinson's disease are typically worse on one side, the participant's report of the more affected side merits recording in the future. Currently the ratings provide evidence of the more impaired extremity. However, the participant can provide longitudinal information about the course of the illness.

The collected data does not indicate the participant's statement of the more affected side. Therefore, correlation of the side of worse performance on the clinical and instrumental assessments cannot be made from the current data. In the future notation will be made about the participant's subjective awareness of the more affected side at the time of the rating session.

*Need for daily life measurements.* The current dataset provides cross-sectional data from in-person structured clinical ratings for specific items that may be abnormal in people with Parkinson's disease and related disorders and corresponding signals from instrumentation on the extremities of the individuals [1,2]. These data were obtained from cross-sectional sessions in the office lasting 30 to 60 min. The instrumentation connected to the participant allowed the participant to sit and stand. The participant could not walk because the extremities were connected to the instrumentation by cords.

Future evaluations may be facilitated by the use of inertial measurement units (IMUs) attached to the extremities that send signals to recording devices. These future developments would facilitate real life continuous monitoring of movements in the office and at home to generate a record of movements throughout the day. Due to the limited motion possible with the current protocol, only an assessment in the office sititng and standing is feasible.

## CRediT author statement

**Timothy P. Harrigan:** Conceptualization, Methodology, Software, Validation, Formal analysis, Investigation, Resources, Data curation, Writing-reviewing and editing, Visualization, Supervision, Project administration

**Brian J. Hwang:** Conceptualization, Methodology, Software, Validation, Formal analysis, Investigation, Resources, Data curation, Writing-reviewing and editing

**Anil K. Mathur:** Validation, Investigation, Resources, Data curation, Writing-reviewing and editing

**Kelly A. Mills:** Investigation, Resources, Data curation, Writing-reviewing and editing

**Alexander Y. Pantelyat:** Investigation, Resources, Data curation, Writing-reviewing and editing

**Jee A. Bang: I**nvestigation, Resources, Data curation, Writing-reviewing and editing

**Alveena B. Syed:** Methodology, Software, Validation, Formal analysis, Investigation, Resources, Data curation, Writing-original draft, Writing-reviewing and editing

**Pankhuri Vyas:** Methodology, Software, Validation, Formal analysis, Investigation, Resources, Data curation, Writing-original draft, Writing-reviewing and editing

**Samuel D. Martin:** Validation, Formal analysis, Investigation, Resources, Data curation, Writing-original draft, Writing-reviewing and editing

**Armaan Jamal:** Validation, Formal analysis, Investigation, Resources, Data curation, Writing-original draft, Writing-reviewing and editing

**Liran Ziegelman:** Investigation, Writing-reviewing and editing

**Manuel E. Hernandez:** Methodology, Software, Validation, Formal analysis, Investigation, Data curation, Writing-original draft, Writing-reviewing and editing

**Dean F. Wong:** Writing-original draft, Writing-reviewing and editing, Supervision

**James Robert Brašić:** Conceptualization, Methodology, Software, Validation, Formal analysis, Investigation, Resources, Data curation, Writing-original draft, Writing-reviewing and editing, Visualization, Supervision, Project administration

## Declaration of Competing Interest

The authors declare that they have no known competing financial interests or personal relationships which have, or could be perceived to have, influenced the work reported in this article.

## Appendix Supplementary materials

Image, application 1

Image, application 2
